# Supplementary material for: Foraging movements are density-independent among straw-coloured fruit bats
Source: R Soc Open Sci. 2020 May 27;7(5):200274. doi: 10.1098/rsos.200274 (PMC7277244; doi:10.1098/rsos.200274)
Supplement: Supplementary Figures [file rsos200274supp1.docx]

**Supplementary Figures**

**Figure S1.** Examples of first passage time selection steps for selecting areas of intensive search (foraging sites). a) Variogram plot: peak of areas of intensive search with a 60 m radius. b) Lavielle segmentation to choose number of clusters to divide the bats trajectory into segments. We selected K=10 segments for the trajectory as there is a clear break in the decrease of the contrast function (JK) after this, JK is the value of the contrast function for the segmented trajectory that allow to choose the appropriate number of segments (see methods section); K is the assumed number of segments in the trajectory. c) Segmented trajectory based on areas of intensive search. Red triangles indicate the beginning and blue triangles the end of each segment.


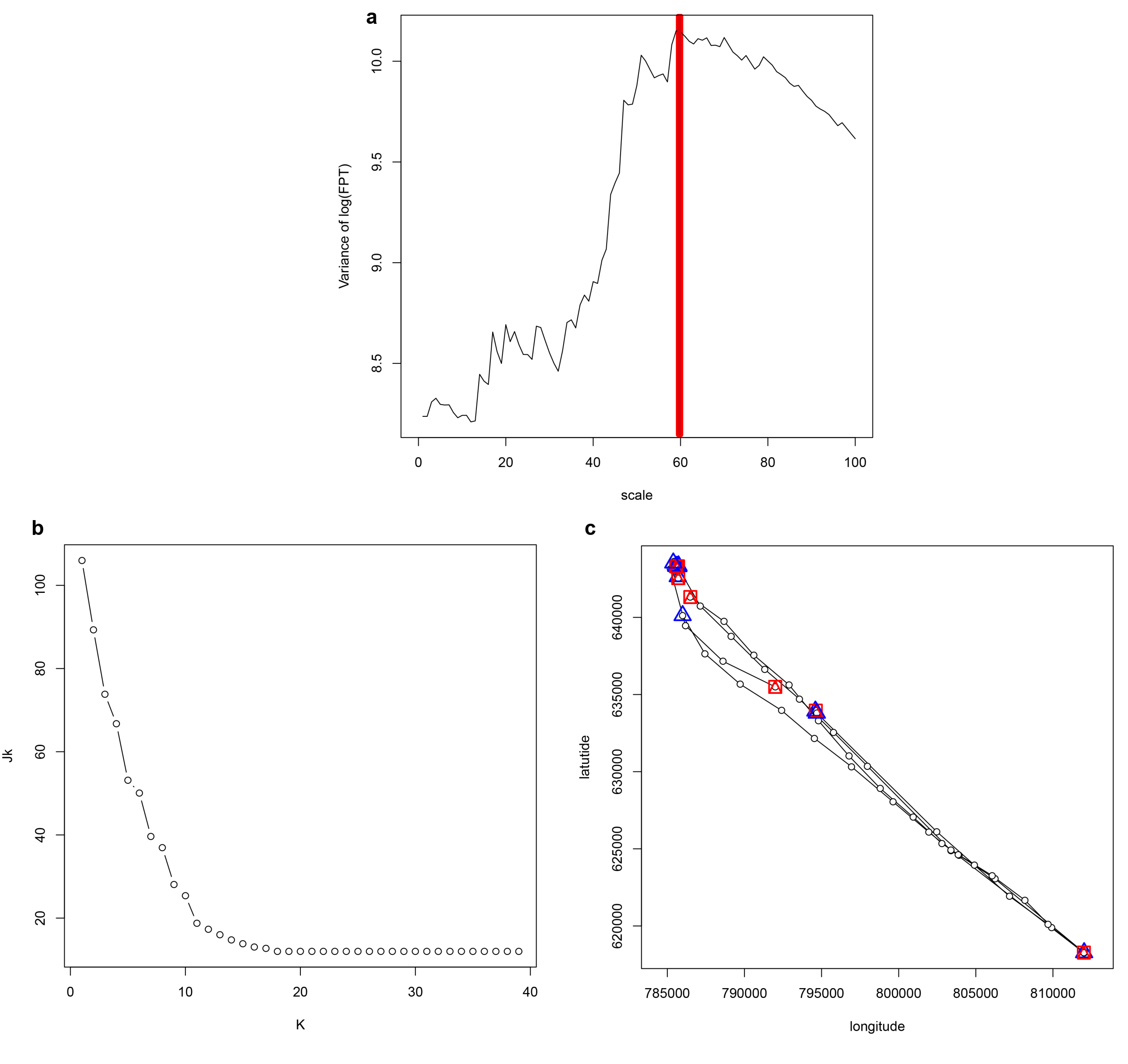


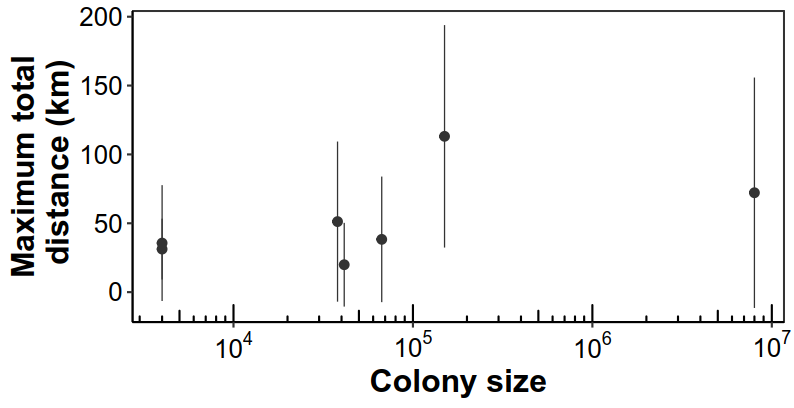
**Figure S2.** Nightly maximum total distance across all colonies of *Eidolon helvum.* Colony size is shown as logarithm base 10. Colony size in number of individuals is as follow: 4.000 (Accra and Kibi low). 38.000 (Ouagadougou low). 41.000 (Kibi high). 67.000 (Ouagadougou high). 150.000 (Accra high). 8.000.000 (Kasanka).

**Figure S3.** Number of foraging sites per night versus the total distance traveled per night.


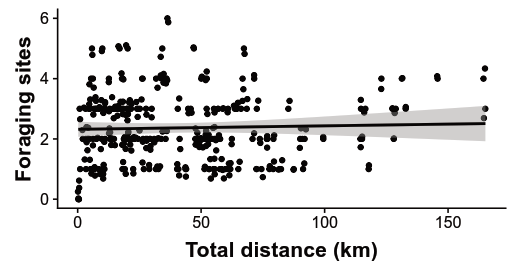


**Figure S4.** Proportion of day roost fidelity and behavioral strategies of *Eidolon helvum*. a. Day roost fidelity proportion; b. Tracks of the bats in Ouagadougou-low; c. Tracks of the bats in Ouagadougou-high; c. Tracks of the bats in Kasanka. Dotted lines: individuals with day roost switching; continuous lines: individuals returning always to the central colony. White triangles: main day roost (central colony); black triangles: alternative day roosts. Abbreviations: AC-low: Accra-low, K-low: Kibi-low, OU-low: Ouagadougou-low, K-high: Kibi-high, OU-high: Ouagadougou-high, AC-high: Accra-high, KA: Kasanka. Differences are only significant (*p-value<0.05) between AC-low and OU-low.


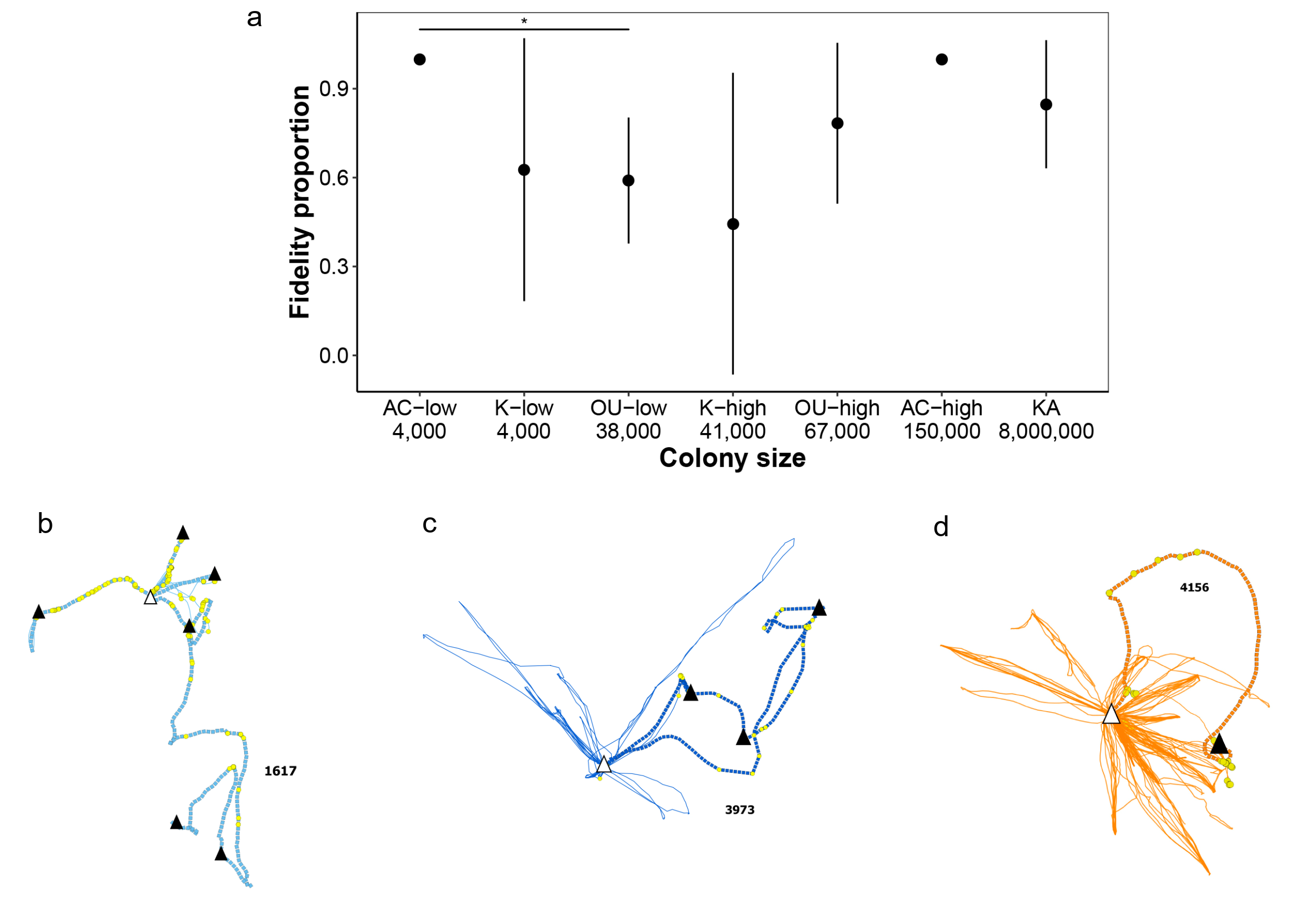


**Supplementary tables**

**Table S1.** GPS and ACC settings. Sex, age, forearm length and body mass are given for each bat used in the analysis. FA = forearm length in mm; BM = body mass in grams. Cohort refers to the different programming GPS schedules. GPS schedule refers to the interval in seconds of the GPS fixes of each logger. Abbreviations: AC-low: Accra-low, K-low: Kibi-low, OU-low: Ouagadougou-low, K-high: Kibi-high, OU-high: Ouagadougou-high, AC-high: Accra-high, KA: Kasanka.

| **Individual** | **Study Area** | **Date tagged** | **Cohort** | **Attachment method** | **GPS on time** | **GPS interval (s)** | **Burst ACC duration (s) (sampling rate Hz)** | **Sex** | **Age** | **FA (mm)** | **BM (g)** |
| --- | --- | --- | --- | --- | --- | --- | --- | --- | --- | --- | --- |
| **1079** | AC-low | 2009-08-25 | 1 | glued | 18:00-6:00 | 600 | 14.09 (18.74) | male | Ad | 118.1 | 284 |
| **1080** | AC-low | 2009-08-25 | 1 | glued | 18:00-6:00 | 600 | 14.09 (18.74) | male | Ad | 113.9 | 244 |
| **1081** | AC-low | 2009-08-25 | 1 | glued | 18:00-6:00 | 600 | 14.09 (18.74) | male | Ad | 123.5 | 274 |
| **1082** | AC-low | 2009-08-27 | 1 | glued | 18:00-6:00 | 600 | 14.09 (18.74) | male | Ad | 120 | 246 |
| **1084** | AC-low | 2009-08-26 | 2 | glued | 18:00-6:00 | 900/300 | 14.09 (18.74) | male | Ad | 115.1 | 239 |
| **1086** | AC-low | 2009-08-26 | 2 | glued | 18:00-6:00 | 900/300 | 14.09 (18.74) | male | Ad | 118.2 | 277 |
| **1088** | AC-low | 2009-08-26 | 2 | glued | 18:00-6:00 | 300 | 14.09 (18.74) | male | Ad | 120 | 247 |
| **697** | AC-low | 2012-07-16 | 2 | glued | 18:00-6:00 | 300 | 14.09 (18.74) | male | Ad | 122,2 | 330 |
| **1607** | AC-high | 2011-02-02 | 3 | glued | 18:00-6:00 | 300/1800 | 14.09 (18.74) | male | Ad | 124.7 | 321 |
| **1608** | AC-high | 2011-02-06 | 3 | glued | 18:00-6:00 | 300/1800 | 14.09 (18.74) | male | Ad | 122,1 | 300 |
| **1610** | AC-high | 2011-02-06 | 3 | glued | 18:00-6:00 | 300/1800 | 14.09 (18.74) | male | Ad | 114,7 | 270 |
| **1612** | AC-high | 2011-02-06 | 3 | glued | 18:00-6:00 | 300/1800 | 14.09 (18.74) | male | Ad | 118,6 | 310 |
| **1613** | AC-high | 2011-02-03 | 3 | glued | 18:00-6:00 | 300/1800 | 14.09 (18.74) | male | Ad | 123,4 | 305 |
| **1615** | AC-high | 2011-02-06 | 3 | glued | 18:00-6:00 | 300/1800 | 14.09 (18.74) | male | Ad | 123,9 | 300 |
| **1616** | AC-high | 2011-02-05 | 3 | glued | 18:00-6:00 | 300/1800 | 14.09 (18.74) | male | Ad | 121 | 292 |
| **1620** | AC-high | 2011-02-06 | 3 | glued | 18:00-6:00 | 300/1800 | 14.09 (18.74) | male | Ad | 119.7 | 255 |
| **1626** | AC-high | 2011-01-02 | 3 | glued | 18:00-6:00 | 300/1800 | 14.09 (18.74) | male | Ad | 119.1 | 280 |
| **1617** | OU-low | 2013-08-10 | 4 | collar | 16:00-6:00 | 150/1800 | 14.09 (18.74) | male | Ad | 118.3 | 265 |
| **1619** | OU-low | 2013-08-19 | 4 | collar | 16:00-6:00 | 150/1800 | 14.09 (18.74) | male | Ad | 118.9 | 275 |
| **1618** | OU-low | 2013-08-19 | 4 | collar | 16:00-6:00 | 150/1800 | 14.09 (18.74) | male | Ad | 103.7 | 250 |
| **1624** | OU-low | 2013-08-21 | 4 | collar | 16:00-6:00 | 150/1800 | 14.09 (18.74) | male | Ad | 124.8 | 280 |
| **1621** | OU-low | 2013-08-27 | 4 | collar | 16:00-6:00 | 150/1800 | 14.09 (18.74) | male | Ad | 124 | 260 |
| **3366** | OU-high | 2014-06-17 | 4 | collar | 16:00-6:00 | 150/1800 | 14.09 (18.74) | male | Ad | 120 | 280 |
| **3967** | OU-high | 2014-06-17 | 4 | collar | 18:00-6:00 | 150/1800 | 14.09 (18.74) | male | Ad | 121.4 | 280 |
| **3971** | OU-high | 2014-06-18 | 4 | collar | 18:00-6:00 | 150/1800 | 14.09 (18.74) | male | Ad | 121.3 | 315 |
| **3969** | OU-high | 2013-04-12 | 4 | collar | 18:00-6:00 | 150/1800 | 14.09 (18.74) | male | Ad | 116.6 | 270 |
| **3970** | OU-high | 2013-05-12 | 4 | collar | 18:00-6:00 | 150/1800 | 14.09 (18.74) | male | Ad | 122.4 | 300 |
| **3972** | OU-high | 2014-06-18 | 4 | collar | 18:00-6:00 | 150/1800 | 14.09 (18.74) | male | Ad | 122 | 255 |
| **3973** | OU-high | 2014-06-18 | 4 | collar | 18:00-6:00 | 150/1800 | 14.09 (18.74) | male | Ad | 123.7 | 265 |
| **1870_1** | K-low | 2011-08-26 | 4 | glued | 18:00-6:00 | 150/1800 | 14.09 (18.74) | male | Ad | 124,6 | 275 |
| **1870_2** | K-low | 2011-08-29 | 4 | glued | 18:00-6:00 | 150/1800 | 14.09 (18.74) | male | Ad | 117,5 | 275 |
| **1875** | K-low | 2011-08-27 | 3 | glued | 18:00-6:00 | 300/1800 | 14.09 (18.74) | male | Ad | 119.1 | 280 |
| **2394** | K-low | 2012-09-09 | 4 | collar | 18:00-6:00 | 150/1800 | 14.09 (18.74) | male | Ad | 118.8 | 275 |
| **2396** | K-low | 2012-08-29 | 4 | collar | 18:00-6:00 | 150/1800 | 14.09 (18.74) | male | Ad | 121.6 | 270 |
| **2402** | K-low | 2012-09-06 | 4 | collar | 18:00-6:00 | 150/1800 | 14.09 (18.74) | male | Ad | 120,3 | 272 |
| **2404** | K-low | 2012-09-13 | 4 | collar | 18:00-6:00 | 150/1800 | 14.09 (18.74) | male | Ad | 121.9 | 270 |
| **2609** | K-low | 2013-09-20 | 4 | collar | 18:00-6:00 | 150/1800 | 14.09 (18.74) | male | Ad | 122.5 | 290 |
| **2608** | K-high | 2013-01-25 | 4 | collar | 18:00-6:00 | 150/1800 | 14.09 (18.74) | male | Ad | 117.9 | 275 |
| **2612** | K-high | 2013-01-25 | 4 | collar | 18:00-6:00 | 150/1800 | 14.09 (18.74) | male | Ad | 125.6 | 250 |
| **2770** | K-high | 2013-01-29 | 4 | collar | 18:00-6:00 | 150/1800 | 14.09 (18.74) | male | Ad | 113,2 | 250 |
| **2772** | K-high | 2013-01-30 | 4 | collar | 18:00-6:00 | 150/1800 | 14.09 (18.74) | male | Ad | 122 | 245 |
| **3359** | KA | 2013-12-04 | 4 | collar | 16:00-6:00 | 150/1800 | 14.09 (18.74) | male | Ad | 126.1 | 290 |
| **3364** | KA | 2013-12-05 | 4 | collar | 16:00-6:00 | 150/1800 | 14.09 (18.74) | male | Ad | 118.1 | 285 |
| **3366** | KA | 2013-12-06 | 4 | collar | 16:00-6:00 | 150/1800 | 14.09 (18.74) | male | Ad | 124,5 | 303 |
| **3369** | KA | 2013-12-04 | 4 | collar | 16:00-6:00 | 150/1800 | 14.09 (18.74) | male | Ad | 120,3 | 321 |
| **3370** | KA | 2013-05-12 | 4 | collar | 16:00-6:00 | 150/1800 | 14.09 (18.74) | male | Ad | 124 | 298 |
| **4148** | KA | 2014-11-29 | 4 | collar | 16:00-6:00 | 150/1800 | 13.2 (20) | male | Ad | 131.2 | 306 |
| **4149** | KA | 2014-11-29 | 4 | collar | 16:00-6:00 | 150/1800 | 13.2 (20) | male | Ad | 116.4 | 278.4 |
| **4150** | KA | 2014-11-29 | 4 | collar | 16:00-6:00 | 150/1800 | 13.2 (20) | male | Ad | 125 | 267 |
| **4151** | KA | 2014-11-29 | 4 | collar | 16:00-6:00 | 150/1800 | 13.2 (20) | male | Ad | 125.4 | 275 |
| **4154** | KA | 2014-11-29 | 4 | collar | 16:00-6:00 | 150/1800 | 13.2 (20) | male | Ad | 122.3 | 269 |
| **4155** | KA | 2014-11-29 | 4 | collar | 16:00-6:00 | 150/1800 | 13.2 (20) | male | Ad | 120.6 | 282 |
| **4156** | KA | 2014-11-29 | 4 | collar | 16:00-6:00 | 150/1800 | 13.2 (20) | male | Ad | 125.1 | 278 |
| **4157** | KA | 2014-11-30 | 4 | collar | 16:00-6:00 | 150/1800 | 13.2 (20) | male | Ad | 126.4 | 315 |
| **4158** | KA | 2014-11-30 | 4 | collar | 16:00-6:00 | 150/1800 | 13.2 (20) | male | Ad | 121.4 | 274 |
| **4159** | KA | 2014-11-29 | 4 | collar | 16:00-6:00 | 150/1800 | 13.2 (20) | male | Ad | 128,3 | 274 |
| **4160** | KA | 2014-11-29 | 4 | collar | 16:00-6:00 | 150/1800 | 13.2 (20) | male | Ad | 127.3 | 286 |
| **4161** | KA | 2014-11-29 | 4 | collar | 16:00-6:00 | 150/1800 | 13.2 (20) | male | Ad | 122.9 | 272 |
| **4162** | KA | 2014-11-30 | 4 | collar | 16:00-6:00 | 150/1800 | 13.2 (20) | male | Ad | 124.6 | 293 |
| **4163** | KA | 2014-11-30 | 4 | collar | 16:00-6:00 | 150/1800 | 13.2 (20) | male | Ad | 121.7 | 273 |
| **4164** | KA | 2014-11-30 | 4 | collar | 16:00-6:00 | 150/1800 | 13.2 (20) | male | Ad | 121.2 | 281 |

**Table S2.** Summary table of tracked nights. foraging distances and foraging sites in different seasons and colonies in *E. helvum*. Abbreviations: AC-low: Accra-low, K-low: Kibi-low, OU-low: Ouagadougou-low, K-high: Kibi-high, OU-high: Ouagadougou-high, AC-high: Accra-high, KA: Kasanka.

**C=**

**A=**

**B=**

| **Individual** | **Colony** | **Seasons** | **Mean total traveled distance (km)** | **sd total traveled distance** | **Maximum foraging distance (km)** | **No. of foraging sites** | **Nights** |
| --- | --- | --- | --- | --- | --- | --- | --- |
| 1079 | AC-low | Wet | 17.1 | 2.3 | 73.2 | 2.6 | 3 |
| 1080 | AC-low | Wet | 12.0 | 1.4 | 3.6 | 2.5 | 2 |
| 1081 | AC-low | Wet | 51.3 | 1.3 | 24.9 | 2.3 | 3 |
| 1084 | AC-low | Wet | 31.0 | 5.0 | 16.0 | 3.0 | 3 |
| 1086 | AC-low | Wet | 62.2 | 8.9 | 36.5 | 2.5 | 2 |
| 1088 | AC-low | Wet | 23.4 | 4.4 | 10.6 | 2.0 | 2 |
| **Mean** |  |  | **32.8** | **3.9** | **27.5** | **2.5** |  |
| **Median** |  |  | **27.2** | **3.4** | **20.5** | **2.5** |  |
| 1607 | AC-high | Dry | 138.0 | 14.9 | 70.3 | 3.0 | 6 |
| 1616 | AC-high | Dry | 71.7 | 1.2 | 31.8 | 2.5 | 3 |
| 1620 | AC-high | Dry | 98.4 | 34.8 | 51.7 | 3.0 | 2 |
| 1626 | AC-high | Dry | 91.0 | 1.3 | 43.3 | 2.0 | 3 |
| **Mean** |  |  | **99.8** | **13.1** | **49.3** | **2.6** |  |
| **Median** |  |  | **98.4** | **13.1** | **49.3** | **2.6** |  |
| 1875 | K-low | Wet | 14.4 | 9.5 | 8.7 | 2.3 | 4 |
| 2394 | K-low | Wet | 27.1 | 6.6 | 17.5 | 3.0 | 2 |
| 2396 | K-low | Wet | 14.5 | 4.1 | 19.2 | 3.0 | 5 |
| 2404 | K-low | Wet | 14.7 | 5.2 | 37.6 | 2.0 | 3 |
| 2609 | K-low | Wet | 31.0 | 3.4 | 15.3 | 2.8 | 5 |
| **Mean** |  |  | **20.3** | **5.8** | **19.7** | **2.6** |  |
| **Median** |  |  | **14.7** | **5.2** | **17.5** | **2.8** |  |
| 2608 | K-high | Dry | 11.5 | 4.9 | 5.2 | 4.0 | 3 |
| 2612 | K-high | Dry | 6.3 | 0.1 | 5.7 | 1.5 | 2 |
| 2772 | K-high | Dry | 21.3 | 21.3 | 15.4 | 5.0 | 2 |
| **Mean** |  |  | **13.0** | **8.8** | **8.8** | **3.5** |  |
| **Median** |  |  | **11.5** | **4.9** | **5.7** | **4.0** |  |
| 1617 | OU-low | Wet low | 69.9 | 18.7 | 39.6 | 2.0 | 4 |
| 1618 | OU-low | Wet low | 7.7 | 7.2 | 22.3 | 3.0 | 5 |
| 1619 | OU-low | Wet low | 26.4 | 20.6 | 25.8 | 2.6 | 6 |
| 1621 | OU-low | Wet low | 35.1 | 12.5 | 25.5 | 2.0 | 3 |
| 1624 | OU-low | Wet low | 20.7 | 5.0 | 20.6 | 3.2 | 5 |
| **Mean** |  |  | **32.0** | **12.8** | **26.8** | **2.6** |  |
| **Median** |  |  | **26.4** | **12.5** | **25.5** | **2.6** |  |
| 3967 | OU-high | Wet high | 11.7 | 11.8 | 12.7 | 1.8 | 5 |
| 3969 | OU-high | Wet high | 18.9 | 14.9 | 16.7 | 2.0 | 5 |
| 3970 | OU-high | Wet high | 5.1 | 0.7 | 3.0 | 3.3 | 4 |
| 3971 | OU-high | Wet high | 27.7 | 14.0 | 28.2 | 2.2 | 6 |
| 3972 | OU-high | Wet high | 50.3 | 29.9 | 39.0 | 3.0 | 2 |
| 3973 | OU-high | Wet high | 24.3 | 13.7 | 34.3 | 2.6 | 5 |
| **Mean** |  |  | **23.0** | **14.2** | **22.3** | **2.5** |  |
| **Median** |  |  | **21.6** | **13.8** | **22.5** | **2.4** |  |
| 3359 | KA | Wet | 30.8 | 30.9 | 53.9 | 1.3 | 6 |
| 3364 | KA | Wet | 52.9 | 15.1 | 49.8 | 3.0 | 5 |
| 3370 | KA | Wet | 67.8 | 35.7 | 47.8 | 2.4 | 5 |
| 4148 | KA | Wet | 60.5 | 1.4 | 28.5 | 1.0 | 5 |
| 4149 | KA | Wet | 70.0 | 29.3 | 44.9 | 1.3 | 7 |
| 4151 | KA | Wet | 54.0 | 2.7 | 26.1 | 2.0 | 7 |
| 4154 | KA | Wet | 38.6 | 19.1 | 38.8 | 1.7 | 7 |
| 4155 | KA | Wet | 18.8 | 4.9 | 17.3 | 2.4 | 7 |
| 4156 | KA | Wet | 80.8 | 42.2 | 54.4 | 1.7 | 7 |
| 4157 | KA | Wet | 63.3 | 3.5 | 28.5 | 2.8 | 6 |
| 4158 | KA | Wet | 48.3 | 5.0 | 20.7 | 3.5 | 6 |
| 4160 | KA | Wet | 52.5 | 20.3 | 36.5 | 1.8 | 6 |
| 4161 | KA | Wet | 28.4 | 18.1 | 29.9 | 1.0 | 3 |
| 4162 | KA | Wet | 5.9 | 5.4 | 4.5 | 2.5 | 2 |
| 4163 | KA | Wet | 7.7 | 5.1 | 37.3 | 2.3 | 8 |
| 4164 | KA | Wet | 97.7 | 44.3 | 55.8 | 2.3 | 6 |
| **Mean** |  |  | **48.6** | **17.7** | **35.9** | **2.1** |  |
| **Median** |  |  | **52.7** | **16.6** | **36.9** | **2.2** |  |

**Table S3.** Generalized linear model predictors for number of foraging sites based on the total distance traveled. Significance terms: ***0.001. **0.01. *0.05.

**C=**

**A=**

**B=**

|  | Total traveled distance~ No. of foraging sites | CI |
| --- | --- | --- |
| Intercept | 2.2^***^ | (1.8. 2.5) |
| slope | 0.005^*^ | (-0.001. 0.01) |
| F_1. 196_ | 2.8 |  |
